# Mapping the Landscape of Brachial Plexus Birth Injury Research: A Comprehensive Bibliometric Study

**DOI:** 10.7759/cureus.52250

**Published:** 2024-01-14

**Authors:** Alexandra F Hoffman, Nathan Khabyeh-Hasbani, Steven M Koehler

**Affiliations:** 1 Orthopedic Surgery, Albert Einstein College of Medicine, Bronx, USA; 2 Orthopedic Surgery, Montefiore Medical Center, Wakefield Campus, Bronx, USA

**Keywords:** brachial plexus injury, bibliometric visualization, peripheral nerves, brachial plexus birth injury, bibliometric analyis

## Abstract

Brachial plexus birth injury (BPBI) is a relatively common condition that poses a significant challenge to children who endure functional impairments later on. This comprehensive bibliometric analysis sought to quantitatively evaluate the existing literature on BPBI, shedding light on authorship, collaboration, publication trends, and keyword analysis to both inform the medical community and foster future research growth. A thorough search of the Web of Science database yielded 712 relevant documents published between 1986 and 2022. The analysis utilized Biblioshiny (K-Synth Srl, Naples, Italy) for bibliometric data, alongside VOSviewer (Centre for Science and Technology Studies, Leiden University, The Netherlands) and TextRazor (TextRazor Ltd., London, UK) for keyword categorization. The literature had an average annual growth rate of 7.94%, with an average document age of 12 years. Collaborative efforts demonstrated 9.6% international co-authorship, with the United States prominently leading global collaborations.

Top producing authors included Yang, Kozin, and Clarke, while the most cited authors were Clarke, Waters, and Curtis. Journals such as the *Journal of Pediatric Orthopedics* and *Plastic and Reconstructive Surgery* emerged as key contributors to the literature. Keyword analysis illuminated prevalent categories like "society" and "health," underscoring the multifaceted nature of BPBI research.

The findings from this bibliometric analysis highlight the dynamic and collaborative landscape of BPBI research, emphasizing the pressing need for continued contributions to address existing gaps in knowledge, enhance global collaboration, and advance the understanding and treatment of this complex condition. Beyond quantitative metrics, this study holds particular significance in its role as a compass for researchers, practitioners, and policymakers invested in BPBI. By offering insights into influential authors, institutions, and emerging trends, this analysis serves as a valuable resource, guiding future research endeavors, fostering interdisciplinary collaboration, and ultimately contributing to improved outcomes for individuals affected by BPBI. The importance of this study lies not only in its informative content but also in its potential to catalyze a collective effort toward refining treatment modalities, promoting preventative measures, and enhancing the overall quality of care for those navigating the challenges of BPBI.

## Introduction and background

Introduction

Brachial plexus birth injury (BPBI) is a relatively common condition, occurring in an estimated 0.5 to 4.6 cases per 1,000 live births [1]. Although it is speculated that many do recover, recent literature has shown that a significant portion of affected children, approximately 8-36%, do not fully recover and experience permanent functional impairments [2, 3]. For such a relatively common condition (the incidence is equal to clubfoot), the field of literature is somewhat sparse [4]. It is important that researchers and physicians contribute to the field to fill gaps in knowledge and advance understanding of the condition. To fill these gaps, we must understand the literature as it stands, who is contributing, and trends in publications [5]. A quantitative way to sort literature is using bibliometric analysis.

Bibliometric analyses have been performed across various specialties and surgical fields [5-8]. A bibliometric analysis is a form of metascience that utilizes statistical analysis designed to assess literature like authorship, publication frequency, collaboration networks, and more [8]. Gaining an overview of the literature is useful because it gives physicians and researchers the ability to understand trends, fill gaps, and enhance collaboration in an objective way [5, 8]. Therefore, the purpose of this analysis was to use bibliometric data to analyze the BPBI literature quantitatively to inform the medical field and encourage future growth.

## Review

Methods

Literature Search

**Figure 1:**
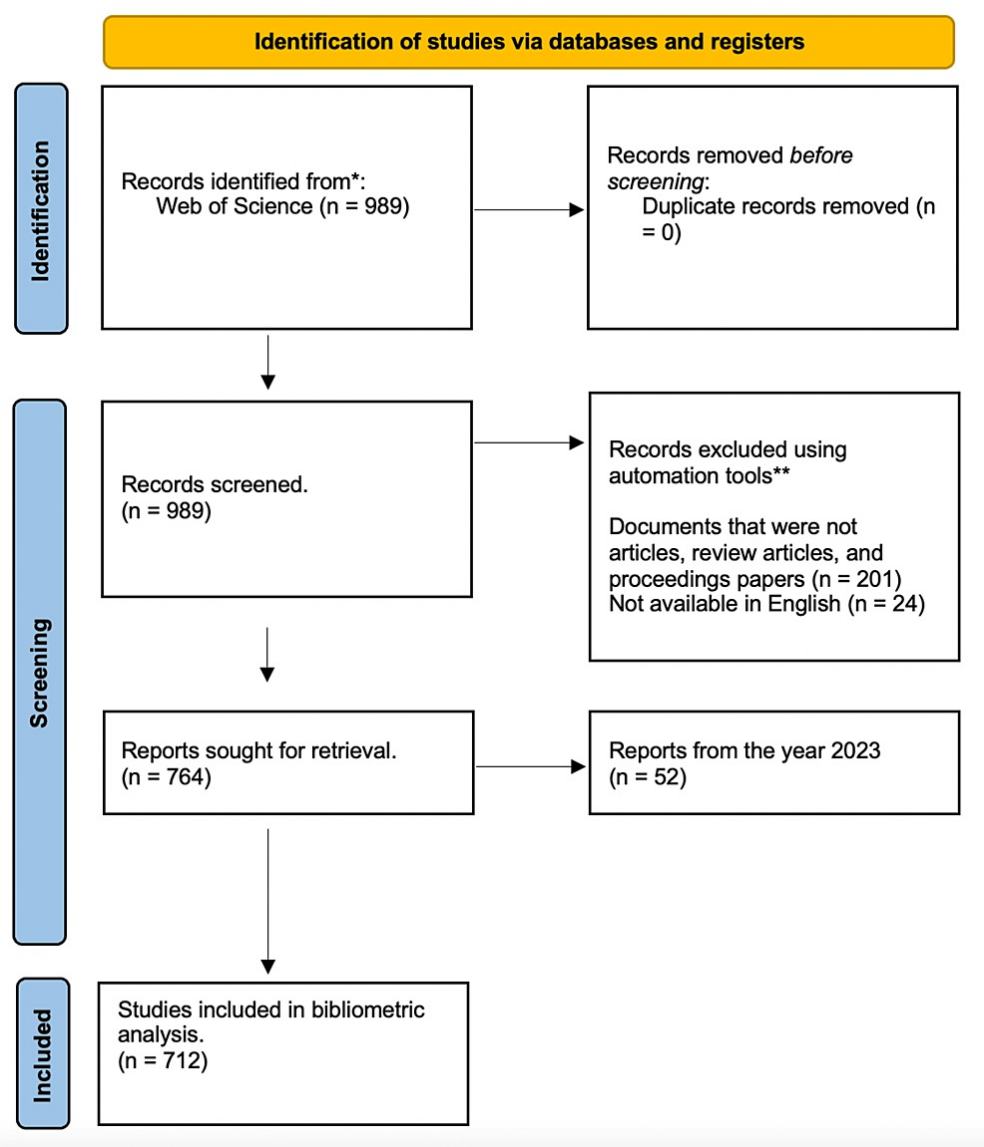
PRISMA flow diagram PRISMA: Preferred Reporting Items for Systematic Reviews and Meta-Analyses

We searched the Web of Science database in November 2023 for articles concerning brachial plexus related birth injuries. The search string was TITLE = (brachial or bpbi) AND (plexus) AND ((neonatal) or (obstetric) or (obstetrical) or (gynecology) or (gynecological) or (birth) or (newborn) or (neonate) or (infant)) AND ((injury) OR (injuries) OR (injury) OR (trauma) OR (lesion) OR (palsy)OR (traumatic) OR (damage)). Documents were included if they were categorized as articles, review articles, or proceeding papers. Articles were included if they were written in English. The search was also narrowed to up until the year 2022 (Figure 1). Although there have been publications in the year 2023, data trend analysis takes place in an annual distribution. Therefore, analytics would be skewed because the full calendar year for 2023 would not be available during the initial document screening.

Bibliometric and Keyword Analysis

Biblioshiny (K-Synth Srl, Naples, Italy) and the ‘Bibliometrix’ R package (K-Synth Srl, Naples, Italy) were used to collect bibliometric data. Data such as annual publication trends, keyword analysis, institutions, geographic data, and co-authorship networks were analyzed [9]. Other variables extracted with Biblioshiny included H-index, impact factor, author-specific demographics, and citation data per article included. VOSviewer [10] (version 1.6.15, Centre for Science and Technology Studies, Leiden University, The Netherlands) was used to create a keyword map to highlight the most prevalent keywords. Keywords were quantified with fractional counting and subsequently used to categorize articles. Fractional counting, which considers the number of keywords, was used to create a more accurate representation of keywords’ impact on the literature [11]. For example, when there were 10 keywords, a keyword received one-tenth of a link. In other words, it weights keywords equally when creating occurrences. In the keyword map‚ the resolution was set to 1.00 to provide the greatest number of clusters that could be visualized at once and separated by color based on what other keywords they were most often linked to. An online platform called TextRazor (TextRazor Ltd., London, UK) was used to extract overarching categories of the keywords pulled from the literature. This allowed us to objectively use artificial intelligence to parse out the most appropriate recurring categories in the literature.

Results

Main Bibliometric Information

**Figure 2:**
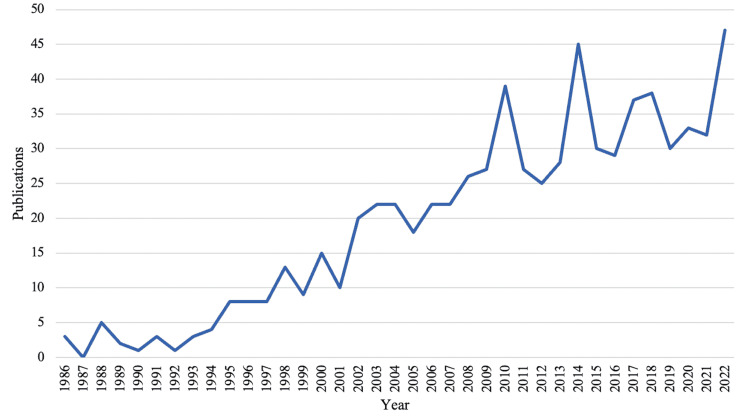
Brachial plexus birth injury (BPBI) publications over time Trends in BPBI publication rates over time. The rate at which the literature has risen was 7.94% as per Biblioshiny calculations.

**Figure 3:**
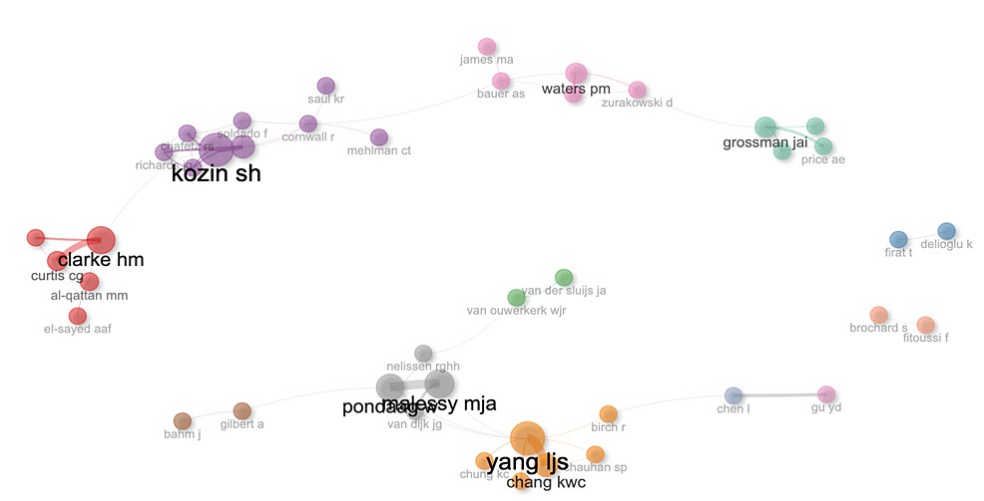
VOSviewer co-authorship network Visual representation of co-authorship networks and author overlap within the field visualized by the VOSviewer program. Larger nodes correlate to higher publication rates and thicker connecting lines correlate with greater overlap between authors.

**Figure 4:**
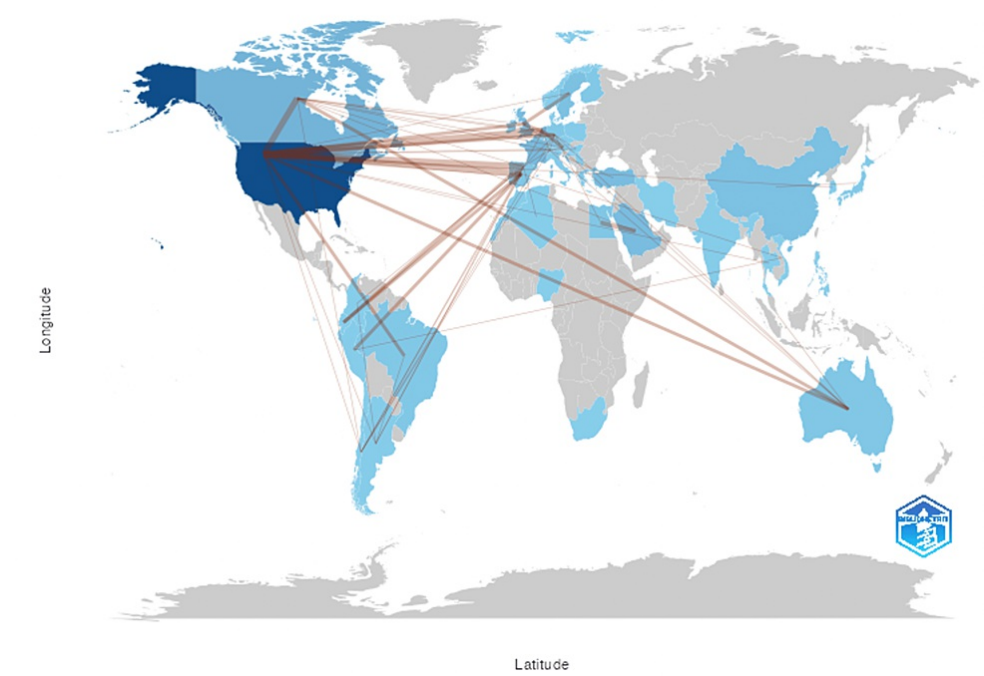
Country collaboration and scientific production Country collaboration and country scientific production heat map. The red lines indicate authorship crossover between different countries on publications. The darker hues of the heat map correlate to higher publication rates. The color grey indicates that no publications were extracted from that location.

Through initial selection, we collected 989 documents. After the criteria were applied, we were left with 712 documents published from 1986 through 2022 from which data was extracted (Figure 1). There were 640 articles, 18 proceedings papers, and 54 reviews. There is an average annual growth rate of 7.94% (Table 1, Figure 2) with a document’s average age being 12 years old. Table 1 shows the main bibliometric information retrieved. There was an average of 22.53 citations per document. There were 1842 unique authors and there was an average of 4.32 authors per document. Furthermore, international co-authorship in this literature was 9.6%. Co-authorship networks are visible in Figure 3, which is a visualization of authors who work together on publications. Biblioshiny code creates these networks by grouping authors into nodes based on their overlap, and links interconnected nodes as well. A greater density link or larger node correlates to greater overlap and more publications respectively. Lastly, country collaboration is listed in Table 1, and mapped in Figure 4. Figure 4 visualizes the links between countries with the red lines and the heat map aspect of Figure 4 visualizes country publication rates which will be discussed later. The United States and the Netherlands had the greatest collaborative efforts amongst any country pairing. The United States also had the greatest overall collaboration rates when compared to other countries.

**Table 1:** Main bibliometric information Main bibliometric information extracted from the literature. This included main information, trends, document content, authors, and collaborations.

Bibliometric Variable	Description	Number (n =)
Main Information		
	Sources (Journals, Books, etc.)	170
	Documents	712
	Annual Growth Rate	7.94%
	Document Average Age (years)	12
	Average Number of Citations per document	22.53
	Total Number of References	7457
Document Contents		
	Author’s Keywords	908
Authors		
	Authors	1842
	Authors of single-authored docs	28
Authors Collaboration		
	Single-authored docs	47
	Co-Authors per Doc	4.32
	International co-authorships	9.55%
Top Country Collaboration		Frequency of Overlap
	USA and Netherlands	14
	USA and France	12
	USA and Canada	10
	USA and Spain	10
	Spain and Portugal	8

Sources, Authors, and H-index

**Figure 5:**
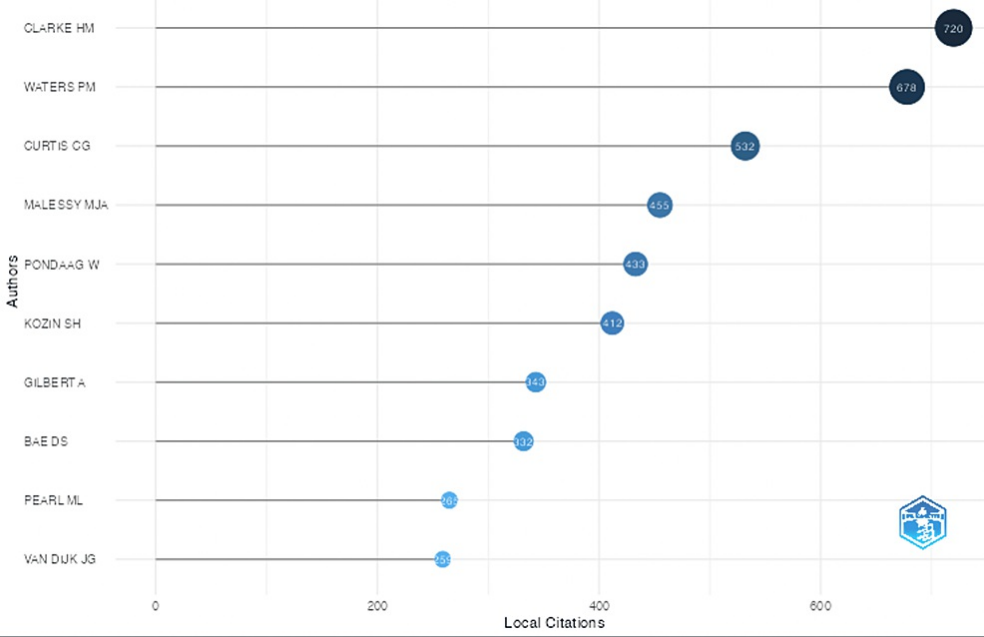
Most locally cited authors These are the most cited authors as returned by Biblioshiny. Local cited authors are the authors who have been cited by the other authors in this literature review. This metric is confined to the citations authors in this study received by other authors included in this study.

**Figure 6:**
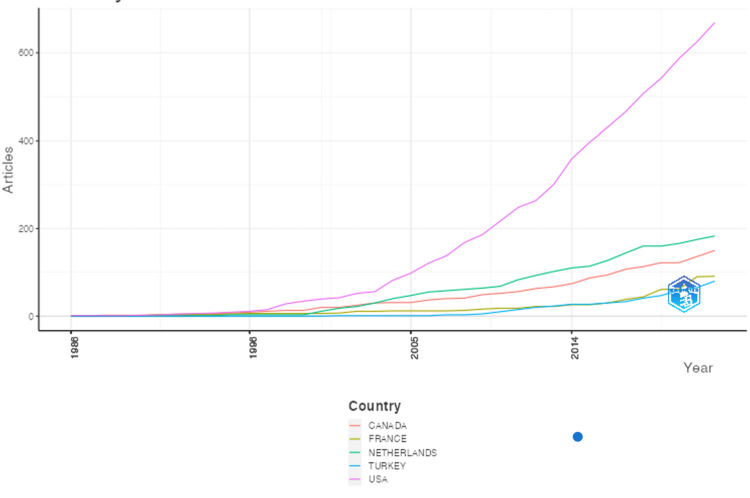
Top country production over time Country publication rates over time indicate the growth within the field and within a country specifically. Beginning in 1986 until the end of 2022, these five countries have had the greatest rise in publication rates over time. The United States had the sharpest rise among these countries.

Table 2 lists the source, author, and h-index information. The top-producing authors are listed in the table, but the top 3 authors of BPBI were Yang, Kozin, and Clarke with approximately 35, 32, and 29 publications identified in this study, respectively. The most locally cited authors were Clarke, Waters, and Curtis (Figure 5), which is the authors most cited by their peers in this literature search. The top producing journals are listed in Table 2, with the 5 highest publishing being the Journal of Pediatric Orthopedics, Journal of Bone and Joint Surgery- American Volume, Journal of Hand Surgery- American volume, Plastic and Reconstructive Surgery, and the Journal of Hand Surgery - European volume with 47, 43, 42, 37, and 29 publications, respectively. Journals with the highest h-index were the Journal of Bone and Joint Surgery-American volume, Plastic and Reconstructive Surgery, and Journal of Bone and Joint Surgery-British volume with h-indices of 25, 21, and 18, respectively. The institutions affiliated with the greatest contribution to the BPBI literature were Leiden University Medical Center, University of Michigan, University of Toronto, Hospital for Sick Children, and Boston Children’s Hospital with their corresponding publishing frequencies listed in Table 3. The countries with the greatest production were the United States, followed by the Netherlands and Canada producing 669, 183, and 150 publications (Table 3). As previously mentioned, Figure 4 visualizes this data with a darker color indicating higher publishing rates. Figure 6 shows the top 5 literature producing countries’ trends in publication over time with the United States publishing rates increasing greatly over time.

**Table 2:** Sources, H-Index, and Authors. This table compiles the “most relevant” sources, authors, and journals with the highest H-index included in the study. The term “most relevant” is the term assigned by Biblioshiny to the top producing journals and authors, or those with the highest publishing rates returned from the data extraction.

Bibliometric Variable	Description	Number (n = )
“Most Relevant” Sources (Journals, Books, etc.)		Publications
	Journal of Pediatric Orthopedics	47
	Journal of Bone and Joint Surgery-American Volume	43
	Journal of Hand Surgery-American volume	42
	Plastic and Reconstructive Surgery	33
	Journal of Hand Surgery-European volume	29
	Journal of Hand Surgery-British and European volume	26
	Developmental Medicine and Child Neurology	23
	Journal of Bone and Joint Surgery-British volume	23
	Childs Nervous System	22
	Journal of Shoulder and Elbow Surgery	20
	Journal of Neurosurgery-Pediatrics	19
	Pediatric Neurology	16
	Microsurgery	13
	Journal of Child Neurology	12
	Journal of Pediatric Orthopedics-part b	12
	Annals of Plastic Surgery	10
	Hand Clinics	10
	Neurosurgery	10
	Muscle & Nerve	9
Top H-index		
	Journal of Bone and Joint Surgery-American Volume	25
	Plastic and Reconstructive Surgery	21
	Journal of Bone and Joint Surgery-British Volume	18
	Journal of Hand Surgery-British and European Volume	17
	Journal of Pediatric Orthopedics	17
	Developmental Medicine and Child Neurology	15
	Journal of Hand Surgery-American Volume	15
	Journal of Hand Surgery-European Volume	11
	Journal of Shoulder and Elbow Surgery	10
	Hand Clinics	9
	Journal of Neurosurgery-Pediatrics	9
	Microsurgery	9
	Journal of Child Neurology	9
	Journal of Pediatric Orthopedics-part b	8
	Annals of Plastic Surgery	8
	Hand Clinics	8
	Neurosurgery	8
	Muscle & Nerve	7
“Most Relevant” Authors		
	Yang Ljs	35
	Kozin Sh	32
	Clarke Hm	29
	Malessy Mja	28
	Pondaag W	26
	Al-Qattan Mm	22
	Chang Kwc	20
	Waters Pm	20
	Curtis Cg	17
	Grossman Jai	17
	Zlotolow Da	16
	Chen L	14
	Cornwall R	14
	Nelissen Rghh	14
	Gu Yd	13
	James Ma	13
	Bauer As	12
	Fitoussi F	12
	Ho Es	12

**Table 3:** Affiliations and country production This table includes the “most relevant” sources and the countries with the highest production of literature. As previously mentioned, “most relevant” refers to the highest publication rates.

Bibliometric Variable	Description	Number (n =)
“Most Relevant” Affiliations		Publications
	Leiden University Medical Center (LUMC)	242
	University of Michigan	158
	University of Toronto	100
	Hospital for Sick Children	51
	Boston Children's Hospital	34
	Vrije Universiteit Amsterdam	32
	Temple University	31
	Harvard University	31
	Cincinnati Children's Hospital Medical Center	29
	Pennsylvania Commonwealth System of Higher Education	29
	King Saud University	27
	Shriners Hospitals for Children Philadelphia	25
	McMaster University	23
Country Scientific Production		
	USA	669
	Netherlands	183
	Canada	150
	France	91
	Turkey	80
	China	63
	United Kingdom	59
	Sweden	58
	Saudi Arabia	51
	Finland	32
	Australia	29
	Egypt	26
	Brazil	24
	Germany	22
	Spain	22
	Italy	19
	Japan	18
	Belgium	17
	Greece/Portugal (tied)	16

Keyword Analysis and Category Extraction

**Figure 7:**
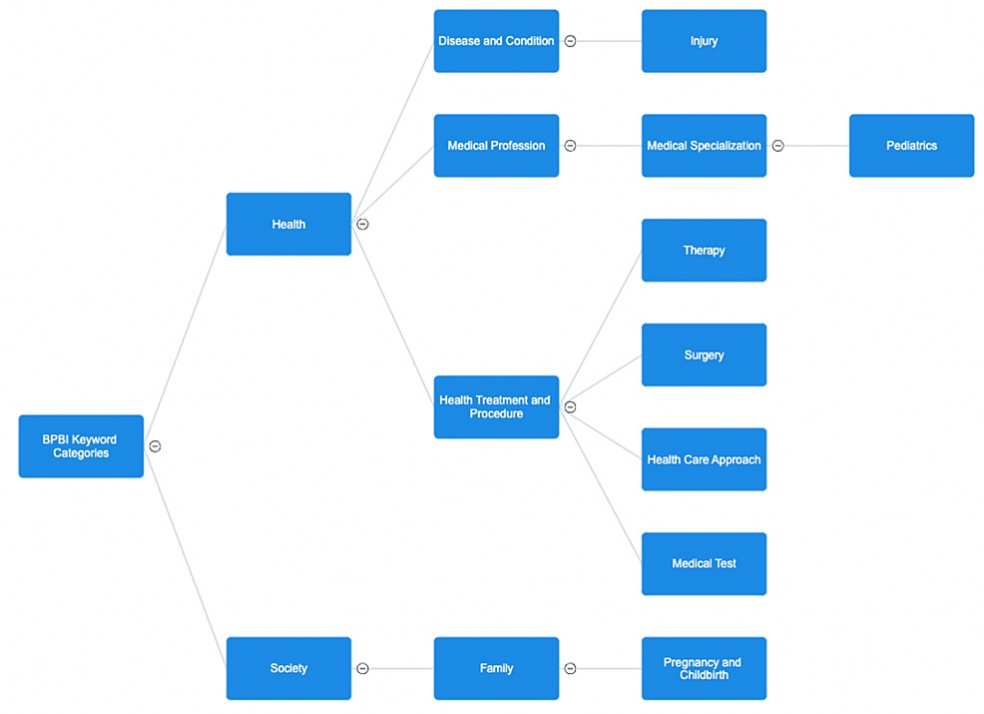
Keyword analysis and categorization Keyword analysis identified these groups and subgroup categorizations within the Brachial Plexus Birth Injury (BPBI) literature. Under BPBI falls health and society, which are then further subdivided within the literature. These specific topics of literature emphasize the importance of treatment modalities, the population affected by BPBI, and other key factors in the field.

There were 908 author’s keywords identified in this analysis. Through keyword analysis using VOSviewer, different categories emerged from within the group when plugged into TextRazor programming. These categories and subcategories are illustrated in Figure 7. Two overarching categories were “society” and “health” from which subcategories branched. Aside from overall categories based on keyword analysis, specific topics were identified from the keywords. These topics included “Brachial plexus injury,” “Erb’s palsy,” “Shoulder joint,” “Human anatomy,” “Nervous system,” “Clinical medicine,” “Traumatology,” “Causes of events,” “Obstetrics,” “arthroscopy,” “Denervation,” “hazards,” and others.

Discussion

Main Bibliometric Data

The BPBI field is growing and expanding (Figure 2). Interestingly, as noted earlier, BPBI has the same incidence as clubfoot (congenital talipes equinovarus) [4, 12]. One would expect that a similar bibliometric study on clubfoot would yield similar results to BPBI, yet, more than double the number of studies have been published on clubfoot [13]. Perhaps this is because in BPBI, treatment methods have rapidly expanded over the past decades and the evolution of surgical methods is largely due to years of research. In this sense, BPBI is a relatively new field in which extensive evolutions in treatment are being implemented [12]. There are many reasons it is important that the literature continues to grow. For example, previous literature has revealed that there is increasing interest and expanding knowledge on BPBI [14]. Therefore, this expanding knowledge, advances in treatment, and methodologies should be published to keep up with the growing field. Contributing to the literature will provide physicians and patients with the most up-to-date information and track BPBI progress over time.

Author Information

As we recognize the top producing authors, it is important to note that this analysis can inform other researchers and physicians of points of reference with long-term experience in the field. Interestingly, the top producing authors are Yang, Kozin, and Clarke, but the most cited authors are Clarke, Waters, and Curtis. This could highlight the varying interests and topics of research within the realm of BPBI. For example, Waters et al. have produced reviews and papers that have created a foundation for the BPBI field [15-17]. Therefore, Waters’ papers may take greater amounts of time to produce, lowering the quantity of production, but increasing citations per paper because they are foundational works. An author may also be more likely to produce greater publications if they spend time at a research-heavy institution than if an author spends most of their time in a clinical setting and has less time to conduct research. These data are important because they can be used as a reference. Authors publishing at higher rates may present the most up-to-date research in the field. If one is looking for foundational information that many rely upon, they can look at the most cited publications.

Collaboration

The number of authors, collaboration amongst authors, and collaboration between countries is also important. Previous literature has revealed that there are inconsistent diagnostic, management, and treatment methods with difficult-to-follow guidelines for patients with BPBI, which may leave physicians at odds with the best treatments available [14]. Our results concerning international author collaboration are promising, but there is room for improvement. We currently show that 9.55% of authorships are internationally co-authored. If this number increased, it would likely lead to more consistent treatment methods for BPBI, more effective evolution of research, and improved patient care [14]. We also created networks of authors based on their authorship overlap (Figure 3). The separate nodes cluster authors that are known to work together. These collaborative efforts may be improved if these author networks could become more interconnected with one another. For example, within the network labelled with Clarke, there are many authors that work together, but this node is not linked to the Yang cluster. If we could establish greater links between separate clusters, communication may allow for unity and consistency.

Collaborative efforts across countries are also important. This differs from single-author international co-authorship because it allows us to see which countries are most interconnected. The United States has the greatest collaboration across the globe with connections to Canada, Europe, South America, and others (Figure 4). Transparency in the field allows treatment methods to build on one another and compound advancements. If the goal is optimizing patient care, then sharing technology and clinical findings across nations will have the greatest impact. Some countries may focus on BPBI more than others if their incidence rates vary, their income, or how important this medical issue is in the greater medical sphere. For example, it is less likely that a country will focus on advancing BPBI treatment if they are battling something like COVID-19 and putting clinical efforts towards developing a lifesaving vaccine. There are many factors to account for when analyzing a country’s contribution to the literature and there are ways to improve it.

Sources and H-index

The top producing resources were the *Journal of Pediatric Orthopedics*, *Journal of Bone and Joint Surgery *- American Volume, *Journal of Hand Surgery *- American Volume, *Plastic and Reconstructive Surgery*, and *Journal of Hand Surgery *- European Volume. This is important because these journals will likely have the greatest store of information on BPBI and would be inferred to have the most up-to-date information on the topic. Researchers may look to submit their work to these journals or use them as primary resources when brushing up on BPBI. These journals in particular focus on a variety of subspecialties, which speaks to the breadth of coverage that is associated with BPBI. Collaboration between pediatrics, orthopedics, plastics, joints, hand, neurology, and more specialties is necessary to further this field and discover treatments. A possible downside to this breadth is that past research has shown that journals outside of neurology do not provide clear, actionable, or consistent information for treating BPBI, which may indicate a need for consolidation or a more directed approach to choosing a journal in the future [14].

H-index is a leading measure for quantifying a researcher’s record impact proposed by Hirsch in 2005 [18, 19]. This value has become an important factor in researchers and institutions securing funding and position applications because it captures the value and productivity of an author [18]. Our study showed that the *Journal of Bone and Joint Surgery *- American Volume, *Plastic and Reconstructive Surgery*, and *Journal of Bone and Joint Surgery *- British Volume had the highest H-index. This information may encourage future researchers to submit their work to these journals because they are productive and may have a greater response rate. A journal with a high H-index may also draw in a greater audience. Authors may also submit their work to these journals because they want to increase their influence within the field and their outreach to influence future research. High productivity is linked to higher H-indices so these journals may also have the most up-to-date information on BPBI. 

Keyword and Categorization Analysis

There is a great paucity of published studies regarding outcomes of BPBI treatment in the field. Of the published studies on BPBI treatment, they are often limited by retrospective nature, small populations, and short timeframes [12]. Keyword categorization and topic extraction revealed that following injury, the social and familial aspects of BPBI are common across the literature. It can be inferred that if society, family, pregnancy, and childbirth are being discussed in the literature, then physicians are focusing on these patients holistically and placing a focus on the mother. This is important in BPBI because familial support is important after these traumatic events and consideration of family may impact the efforts towards preventative measures to protect the mother and child. Treatment through “surgery,” “health care approaches,” “medical tests,” and “therapy” underlies the goal of improving on current treatment methods while discovering others that may be just as beneficial. Furthermore, the category of medical specialization and pediatrics emphasizes the importance of this field. There are often statistics emphasizing the lack of pediatric specialists and these documents may be focused on the specific role of these types of physicians, as well as the need to focus on such a vulnerable patient population.

Limitations

The field of BPBI is relatively small [5]. Although this gave us the ability to analyze the data that is available more readily, it is still a small sample. In the future, we could expand our method to other sources like media, magazines, and websites, or increase our number of databases to address this. Furthermore, we are limited by the decision of whether to include the year 2023 in analyses. This decreased the sample size and may have discounted prominent literature that was published this year, but it was necessary to accurately track trends from 1986 until 2022 without negatively skewing due to a full year of research being cut short. Another potential limitation is that only one database was used. However, it should be noted that Web of Science includes more than 21,517 journals, provides expansive coverage through a comprehensive content collection, and uses artificial intelligence to scrape the web for publications [5]. Furthermore, we did not categorize and identify the specific topic of each article. This would be a target of future research. Lastly, an area of future exploration could analyze how generative artificial intelligence impacts publishing trends and publishing regulations as technology evolves. 

## Conclusions

In conclusion, this comprehensive bibliometric analysis provides a rich source of information for researchers, practitioners, and policymakers interested in BPBI. The collaborative and global nature of research, coupled with the enduring impact of the literature, suggests a vibrant and dynamic field that continues to evolve. Researchers and physicians can use this analysis to address gaps in the field and to find the most impactful authors and institutions to reference in their own practice and collaborate with in advancing the treatment and prevention of BPBI.
